# Isolation of an Extract from the Soft Coral Symbiotic Microorganism *Salinispora arenicola* Exerting Cytoprotective and Anti-Aging Effects

**DOI:** 10.3390/cimb44010002

**Published:** 2021-12-22

**Authors:** Xanthippi P. Louka, Aimilia D. Sklirou, Géraldine Le Goff, Philippe Lopes, Eleni-Dimitra Papanagnou, Maria S. Manola, Yehuda Benayahu, Jamal Ouazzani, Ioannis P. Trougakos

**Affiliations:** 1 Department of Cell Biology and Biophysics, Faculty of Biology, National and Kapodistrian University of Athens, 15784 Athens, Greece; xanthiluka@gmail.com (X.P.L.); asklirou@biol.uoa.gr (A.D.S.); epapanagnou@biol.uoa.gr (E.-D.P.); mmanola@biol.uoa.gr (M.S.M.); 2 CNRS, Institut de Chimie des Substances Naturelles, UPR 2301, 91190 Gif-sur-Yvette, France; geraldine.legoff@cnrs.fr (G.L.G.); Philippe.LOPES@cnrs.fr (P.L.); Jamal.Ouazzani@cnrs.fr (J.O.); 3 School of Zoology, George S. Wise Faculty of Life Sciences, Tel Aviv University, Ramat Aviv, Tel Aviv 69978, Israel; yehudab@tauex.tau.ac.il

**Keywords:** aging, antioxidant responses, marine microorganism, proteostasis network, *Salinispora arenicola*

## Abstract

Cells have developed a highly integrated system responsible for proteome stability, namely the proteostasis network (PN). As loss of proteostasis is a hallmark of aging and age-related diseases, the activation of PN modules can likely extend healthspan. Here, we present data on the bioactivity of an extract (SA223-S2BM) purified from the strain *Salinispora arenicola* TM223-S2 that was isolated from the soft coral *Scleronephthya lewinsohni*; this coral was collected at a depth of 65 m from the mesophotic Red Sea ecosystem EAPC (south Eilat, Israel). Treatment of human cells with SA223-S2BM activated proteostatic modules, decreased oxidative load, and conferred protection against oxidative and genotoxic stress. Furthermore, SA223-S2BM enhanced proteasome and lysosomal-cathepsins activities in *Drosophila* flies and exhibited skin protective effects as evidenced by effective inhibition of the skin aging-related enzymes, elastase and tyrosinase. We suggest that the SA223-S2BM extract constitutes a likely promising source for prioritizing molecules with anti-aging properties.

## 1. Introduction

Aging is characterized by a time-dependent decline in functional capacity and stress resistance, as well as by increased mortality and morbidity rates [1]. Aging is not just a passive process, as it is regulated by evolutionary conserved molecular and cellular signaling events [2,3]. Organisms are constantly challenged by both internal (metabolic-related) and external (diet- or environmental-derived) stressors, which can lead to the gradual accumulation of damaged biomolecules and eventually compromise cellular homeostasis [4,5]. Proteins are involved in all pivotal cellular functions and therefore, proteome homeostasis (proteostasis) is critical for cellular functionality and survival [6].

Proteome integrity is maintained by the proteostasis network (PN), which extends to all subcellular compartments and is regulated at the cellular, tissue and organismal level in order to ensure organismal health and longevity [7]. Sustaining proteostasis requires cells to coordinate and regulate the functions of synthesis, folding, sorting, transport, and degradation of all polypeptides [8,9]. These cellular functions are mostly carried out by three major interconnected branches of PN, namely the network of molecular chaperones, the ubiquitin proteasome-(UPP), and autophagy lysosome-(ALP) proteolytic pathways [10,11].

Molecular chaperones ensure proper protein folding and conformational maintenance, and they also cooperate with the degradation pathways [12]. UPP is the main site of protein synthesis’ quality control and is involved in the recycling of normal short-lived proteins and of non-repairable misfolded or unfolded proteins [6], while ALP is responsible for the recognition and removal of protein aggregates and damaged organelles [13]. Loss of proteostasis is a hallmark of aging [14], as well as a major risk factor for many age-related human pathologies, including cancer, diabetes, cardiovascular disorders, and neurodegenerative diseases [15]. Therefore, PN activation is considered a promising approach for delaying the onset and/or progression of aging and age-related diseases [16,17,18].

Aging of the skin is characterized by structural and functional changes in (among others) extracellular matrix components, such as elastin [19]. Elastase is the enzyme involved in the degradation of elastin, resulting in the development of wrinkles and loss of elasticity [20]. Tyrosinase is a key enzyme in melanin synthesis, which is responsible for the pigmentation of (among others) human skin [21,22]. However, the excessive activation of tyrosinase is associated with several skin disorders, such as hyperpigmentation and melanoma [22,23]. Therefore, elastase and tyrosinase inhibition constitutes a prominent approach against skin aging.

Natural products (NPs) have a long history—well documented, before the birth of modern science and technology, in various cultures—of treating human diseases [24,25,26]. Thus, NPs can serve as highly enriched starting materials and scaffolds for drug development; indeed, over half of all drugs are either NPs, NPs-derived, or NPs-inspired [27,28,29]. The marine world, that harbors the most biodiversity, is largely unexplored and therefore may be the vastest resource to discover novel NPs [30]. Among the organisms that live in marine environments, soft corals are promising providers of bioactive NPs and are known to host many and diverse microorganisms [31,32].

Given these facts, we are currently performing an extensive high-throughput screening of NPs to identify bioactive molecules that could potentially delay cellular senescence and/or in vivo aging. In the study presented herein, we examined a methanolic extract, namely SA223-S2BM, of the *Salinispora arenicola* (*S. arenicola*) TM223-S2 strain by applying in vitro assays, as well as cell-based and in vivo bio-guided approaches. *S. arenicola* TM223-S2 strain was isolated from the soft coral *Scleronephthya lewinsohni* (*S. lewinsohni*) (initially named as *Scleronephthya lewinsohni* by Verseveldt and Benayahu, 1978), which was sustainably obtained from the mesophotic Red Sea ecosystem EAPC (south Eilat, Israel) at a depth of 65 m. The SA223-S2BM methanolic extract activated cytoprotective mechanisms both in human cells and *Drosophila melanogaster* (*D. melanogaster*) *flies* and exerted in vitro skin protective properties. Therefore, the SA223-S2BM extract could be the source for the likely identification of molecules with potential cytoprotective and anti-aging properties.

## 2. Materials and Methods

### 2.1. SA223-S2BM Extract’s Origin and Isolation

#### 2.1.1. Coral Collection

The soft coral *S. lewinsohni* was collected by an ROV H800 (ECA Robotics) operated by Sam Rothberg R/V of the Interuniversity Institute for Marine Sciences in Eilat (IUI). In situ photography was carried out by a low-light black and white camera VS300 (ECA Robotics) and 1CAM Alpha HD camera (SubC Imaging). Samples were obtained by the ROV arm from the mesophotic reef of Eilat, off the oil jetty terminal (29°30′47.09″ N, 34°35′56.71″ E), at 65 m, on 7 March 2017. The collection site is characterized by a moderate slope with rocky patches surrounded by a sandy seabed. Upon collection, the material was immediately frozen at −80 °C. A voucher specimen was preserved in 70% ethanol for taxonomic identification and deposited at the Steinhardt Museum of Natural History, Tel Aviv University (SMNHTAU_Co_37500).

#### 2.1.2. Strain Isolation and Identification

*S. arenicola* TM223-S2 was isolated from a 1 mL sample of *S. lewinsohni*. The collected soft coral sample was ground in sterile seawater and heated at 50 °C for 1 h. The resulting suspension was serially diluted, plated on selective isolation agar medium, and incubated at 28 °C for at least 6 weeks. The *Salinispora* strain was isolated, and the colony was purified on marine broth agar (MBA) (Difco, Thermo Fisher Scientific Inc., Waltham, MA, USA) and conserved at −20 °C in a 10% glycerol solution.

#### 2.1.3. Phylogeny Investigation

Phylogenic analysis was performed using a fragment of the 16S rRNA gene amplified from the genomic DNA of *S. arenicola* TM223-S2. Genomic DNA of the strain TM223-S2 was isolated using a DNeasy Blood and Tissue Kit (Qiagen), according to the manufacturer’s instructions. The 16S ribosomal RNA fragment was amplified by PCR using primers 16S F 27, AGA GTT TGA TC(AC) TGG CTC AG (Tm: 56.3 °C), and 16S R 1492, TAC GG(CT) TAC CTT GTT ACG ACT T (Tm: 57.5 °C). Amplicons were sequenced by Sanger sequencing and the sequences were aligned against the 16S ribosomal RNA database of the Targeted Loci project of NCBI using MUSCLE. The alignment was manually inspected, and gaps were removed. The evolutionary history was inferred by using the maximum likelihood method and Tamura–Nei model [33] with 1000 replicates. The initial tree for the heuristic search was obtained automatically by applying neighbor-join and BioNJ algorithms to a matrix of pairwise distances estimated using the maximum composite likelihood (MCL) approach, and then selecting the topology with a superior log likelihood value. Evolutionary analyses were conducted in MEGA X version 10.1.7 [34].

#### 2.1.4. Cultivation Conditions

*S. arenicola* TM223-S2 spores were conserved at −20 °C in 10% glycerol. Before cultivation, the strain was revived for 15 days on a 15 cm Petri plate containing MB (Difco, Thermo Fisher Scientific Inc., Waltham, MA, USA) supplemented with 2% Agar (Difco, Thermo Fisher Scientific Inc., Waltham, MA, USA). Sterile seawater (4 × 10 mL) was poured on the plate surface, and the spores and mycelium were recovered from the plate by gentle scratching of the surface with a scalpel to generate the inoculum. Liquid-state fermentation (LSF) was conducted in a 2 L Erlenmeyer flask for 20 days at 28 °C and 130 rpm agitation. Amberlite XAD16 resin (30 g/L) (Amberlite XAD16HP N, Certis, Lenntech, Delfgauw, The Netherlands) was added prior to sterilization to allow the in situ trapping of the microbial metabolites. Five milliliters of the inoculum were used to inoculate 1 L on MB containing 30 g of Amberlite XAD16 resin. Agar-state fermentation coupled with solid-phase extraction (SPE) [35] was performed on MB medium (Difco, Thermo Fisher Scientific Inc., Waltham, MA, USA) supplemented with 2% Agar (Difco, Thermo Fisher Scientific Inc., Waltham, MA, USA) over 20 days at 28 °C. Sterilized Amberlite XAD16 (35 g) mixed with 35 mL of inoculum was homogeneously spread on the surface of 1 MB agar Petri plate (25 cm × 25 cm).

For LSF, the XAD16 resin was separated from the broth culture via filtration and washed with water before being eluted with ethyl acetate (100 mL) and then with methanol (100 mL). The eluates were concentrated to dryness in vacuo and solubilized in DMSO at 10 mg/mL concentration. The resulted extract, SA223-S2LAE (extracted using ethyl acetate), was used for biological evaluations.

For SPE, the XAD16 resin was recovered by carefully scratching the agar plate surface. The recovered resin was washed with water to eliminate the biomass before being eluted with ethyl acetate (100 mL) and then with methanol (100 mL). The eluates were concentrated to dryness in vacuo and solubilized in DMSO at 10 mg/mL concentration. The resulted extracts, SA223-S2BAE (extracted using ethyl acetate) and SA223-S2BM (extracted using methanol), were used for biological evaluations.

### 2.2. Elastase and Tyrosinase Inhibitory Activities

The elastase inhibitory activity of the prioritized extract was evaluated using elastase from porcine pancreas (PPE) type IV (Merck KGaA, Darmstadt, Germany) and the substrate N-succinyl-Ala-Ala-Ala-p-nitroanilide (Merck KGaA, Darmstadt, Germany), as previously described [36]. The capacity of the extract to inhibit the catalytic action of tyrosinase in the oxidation of L-DOPA to dopachrome was determined, as previously reported [37].

### 2.3. Cell Lines, Cell Culture Conditions and Cell Viability Assay

Human foreskin fibroblasts (BJ cells) were purchased from the American Tissue Culture Collection (ATCC), while human immortalized keratinocytes (HaCaT cells) were obtained from Elabscience, Inc. Both cell lines were cultured in Dulbecco’s Modified Eagle’s Medium (DMEM) (Thermo Fisher Scientific Inc., Waltham, MA, USA) supplemented with 10% (*v*/*v*) fetal bovine serum (FBS) and 1% (*v*/*v*) non-essential amino acids and were maintained in conditions of 5% CO_2_, 95% humidity and 37 °C. In all experimental procedures applied, cells were subcultured by using a trypsin/EDTA solution (Thermo Fisher Scientific Inc., Waltham, MA, USA). The effect of the SA223-S2BM extract on cell viability was estimated using the MTT assay, as previously reported [38]. Cell treatment with hydrogen peroxide (H_2_O_2_) and doxorubicin (DXR) was performed as described in figure legends; H_2_O_2_ and DXR were obtained from Merck KGaA (Darmstadt, Germany).

### 2.4. Drosophila Lines and Longevity Assay

In this study, we used the *Drosophila* w^1118^ strain purchased from Bloomington *Drosophila* Stock Center (Bloomington, IN, USA). Flies were maintained at 25 °C and 60% humidity on a 12 h light: 12 h dark cycle. In all presented experiments, only somatic tissues (head and thorax) of flies were analyzed, since reproductive tissues display distinct regulation of proteostatic mechanisms and aging rates as compared to somatic tissues [39].

The longevity of flies was assayed as described before [40] by using female and male flies (equal number per sex) that were cultured in vials containing (or not) the studied extract. Flies were transferred to vials with fresh food every two days and deaths were scored daily. For survival curves and statistical analysis, the Kaplan–Meier procedure and log-rank (Mantel–Cox) test were used; significance was accepted at *p* < 0.05.

### 2.5. Measurement of Reactive Oxygen Species (ROS)

Cells or flies’ tissues were incubated with the CM-H_2_DCFDA dye (Thermo Fisher Scientific Inc., Waltham, MA, USA) at a concentration of 10 μM in PBS for 30 min in the dark (37 °C and 25 °C, respectively). Following dye removal, cells or flies’ tissues were incubated with PBS for 15 min in the dark (37 °C and 25 °C, respectively), lysed in lysis buffer [150 mM NaCl, 1% (*v*/*v*) NP-40, 50 mM Tris, pH 8.0] and, then centrifuged at 14,000 g for 15 min (4 °C). Afterwards, protein content was measured with the Bradford assay (Bio-Rad Laboratories, Inc., Hercules, CA, USA) and the produced fluorescence was measured in a Spark^®^ multimode microplate reader (Tecan Trading AG, Männedorf, Switzerland) at excitation and emission wavelengths of 490 and 540 nm, respectively. Hydrogen peroxide (H_2_O_2_) was used as a positive control, as it is widely known to cause oxidative damage/stress [41].

### 2.6. RNA Extraction, cDNA Synthesis and Quantitative Real-Time PCR (Q-RT-PCR) Analysis

Total RNA was extracted from cells using RNAiso plus (Takara Bio Inc., Shiga, Japan) and quantified with a BioSpec-nano spectrophotometer (Shimadzu Inc., Kyoto, Japan). Subsequently, 1 μg RNA was converted to cDNA using the FastGene Scriptase II cDNA Kit (NIPPON Genetics, Düren, Germany). To carry out Quantitative Real-Time PCR analyses, the 5x HOT FIREPol^®^ EvaGreen^®^ qPCR Supermix (Solis BioDyne, Tartu, Estonia) and PikoReal^TM^ Real-Time PCR System (Thermo Fisher Scientific Inc., Waltham, MA, USA) were used. Specific primers for the human genes studied were as described before [42]. The *HMBS* gene was used as a normalizer.

### 2.7. Immunoblotting Analysis and Measurement of Proteasome and Cathepsins Activity

Immunoblotting studies were conducted as previously described [43]. Primary and horseradish peroxidase-conjugated secondary antibodies were applied for 1 h at room temperature and immunoblots were developed using an enhanced chemiluminescence reagent kit (Clarity^TM^ Western ECL Substrate, Bio-Rad Laboratories, Inc., Hercules, CA, USA). Immunoblots quantitation was performed by scanning densitometry and ImageJ software [44].

Proteasome and cathepsins enzymatic activities in cells or flies’ tissues were measured at a VersaFluor™ Fluorometer System (Bio-Rad Laboratories, Inc., Hercules, CA, USA) using Suc-LLVY-AMC (chymotrypsin-like activity, CT-L) and Z-FR-AMC fluoropeptides (Enzo Life Sciences, Inc., Farmingdale, NY, USA), respectively, as previously described [42,45,46]. 6-bromoindirubin-3′-oxime (6BIO) was used as a positive control, as it is known to activate UPP both in human cells and *Drosophila* flies [42,47]; the drug Rapamycin, was used as a positive control for ALP and cathepsins induction [48].

### 2.8. Antibodies Used

Primary antibodies against the β5 (sc-55009) proteasome subunit, NQO1 (sc-16464), Beclin 1 (BECN1) (sc-11427), Clusterin (CLU) (sc-6419), HSP27 (sc-13132), HSP70 (sc-59570), and GAPDH (sc-25778), as well as the horseradish peroxidase-conjugated secondary antibodies, were purchased from Santa Cruz Biotechnology, Inc. (Dallas, TX, USA). The primary antibodies against 53BP1 (4937), phospho-Histone H2A.X^Ser139^ (γH2A.X) (9718) and phospho-p53^Ser15^ (9286) were obtained from Cell Signaling Technology, Inc. (Danvers, MA, USA). The antibody against SQSTM1/p62 (BML-PW9860) was purchased from Enzo Life Science, Inc. (Farmingdale, NY, USA).

### 2.9. Statistical Analyses

Experiments were performed at least in duplicates. MS Excel was used for the statistical analyses. Statistical significance was evaluated using Student’s *t*-test (*t*-test). Data points correspond to the mean of the independent experiments and error bars denote the standard deviation (SD); significance at *p* < 0.05 or *p* < 0.01 is indicated in graphs by one or two asterisks, respectively.

## 3. Results

### 3.1. Identification of the Actinomycete Associated with the Soft Coral S. lewinsohni

The targeted microbial strain was isolated from the coral *S. lewinsohni*, which is a soft coral endemic to the Red Sea. The colonies of this coral are typically found in deep sites, both in the upper mesophotic coral reef ecosystems (30–60 m) and in the lower mesophotic coral reef ecosystems (60–105 m) [49]. Moreover, it is an azooxanthellate soft coral that obtains its energetic requirements by filter feeding and, therefore, it is found in habitats exposed to strong water currents.

**Figure 1:**
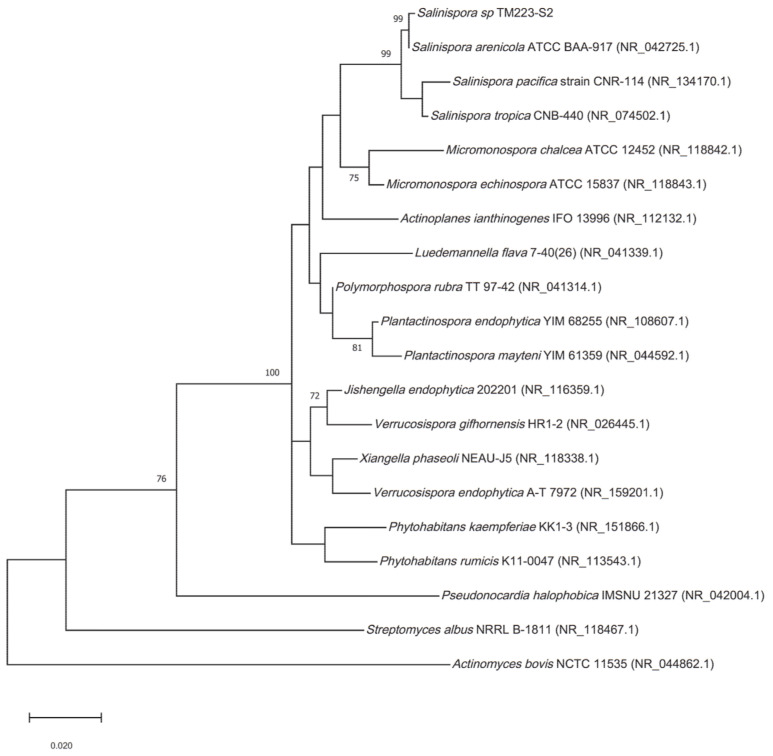
Phylogenic tree. Maximum-likelihood tree obtained from 16S rRNA sequence alignment of the isolate TM223-S2 and representatives of the three *Salinispora* spp. described, together with relatives from Actinomycetales. Only bootstrap values greater than 70% are reported (1000 replicates). Genbank accessions are mentioned in brackets.

From the phylogenetic analysis based on the 16S rRNA sequence, the isolate TM223-S2 was found to belong to the *Salinispora* genus (Figure 1).

Since *S. arenicola* can be distinguished from *S. pacifica* and *S. tropica* based on the 16S rRNA sequence [50], we named this isolate *S. arenicola* TM223-S2 with a Genbank accession number MW446171. The strain was cultivated as reported in the Materials and Methods Section and extracted respectively by ethyl acetate and methanol. Preliminary biological investigation indicated that the extract from the liquid culture, SA223-S2BAE, and the ethyl acetate extract from the solid culture, SA223-S2LAE, were devoid of any significant biological activity (Figure S1). Therefore, only the methanolic extract from the solid culture associated to solid-phase extraction, SA223-S2BM, was used in this study.

### 3.2. The Extract SA223-S2BM Shows no Toxicity in Human Cells and Activates Key Components of the PN

**Figure 2:**
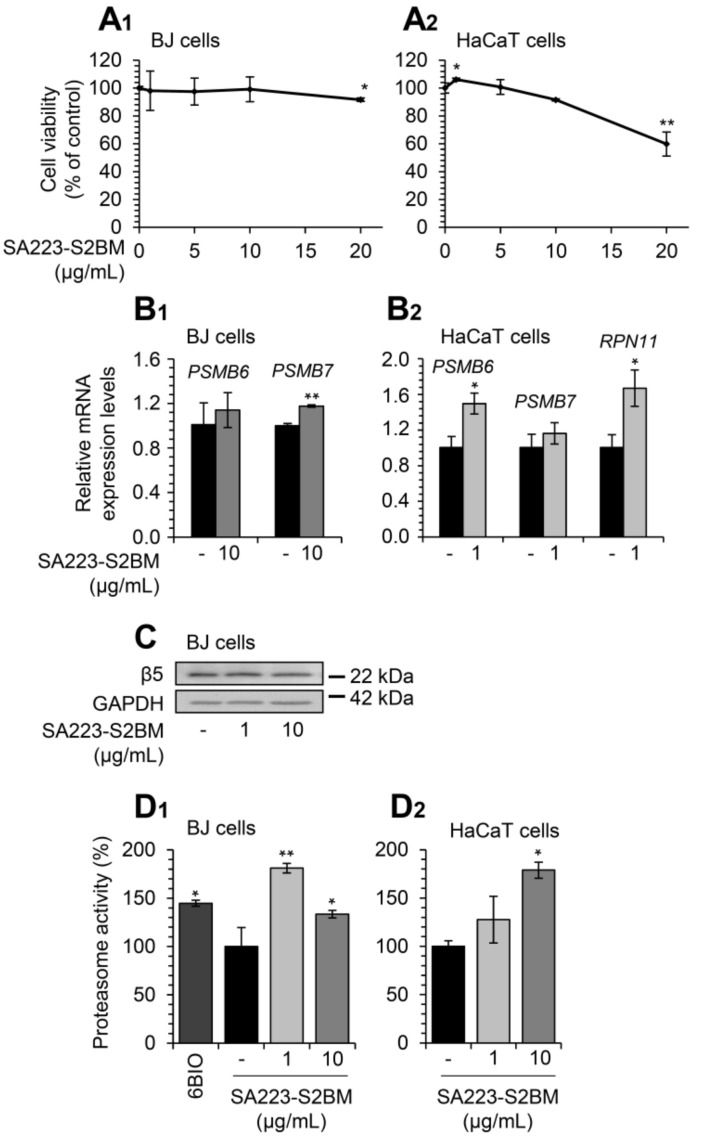
The extract SA223-S2BM exerts no toxicity in human normal skin fibroblasts and immortalized keratinocytes and activates UPP. (**A_1_**,**A_2_**) Relative (%) survival levels (MTT assay) of BJ skin fibroblasts (**A_1_**) and HaCaT keratinocytes (**A_2_**) exposed to the indicated concentrations of the SA223-S2BM extract for 24 h. (**B_1_**) Relative expression levels of 20S (*PSMB6, PSMB7)* proteasome genes in BJ cells after incubation with 10 μg/mL of the SA223-S2BM extract for 24 h. (**B_2_**) Relative expression levels of 20S (*PSMB6, PSMB7*) and 19S (*RPN11*) proteasome genes in HaCaT cells after treatment with 1 μg/mL of the extract for 24 h. (**C**) Representative immunoblotting analysis of the 26S proteasome subunit β5 expression levels after a 24 h exposure of BJ cells to the shown concentrations of the extract. (**D_1_**,**D_2_**) Relative (%) chymotrypsin-like proteasome peptidase activity in BJ (**D_1_**) and HaCaT (**D_2_**) cells treated with the indicated concentrations of the SA223-S2BM extract for 24 h. The compound 6BIO (2 μM) was used as a positive control. The *HMBS* gene expression (**B_1_**,**B_2_**) and GAPDH probing (**C**) were used as reference for RNA and protein input, respectively. Quantitation of shown blots is presented in Figure S2. In (**A_1_**,**A_2_**,**D_1_**,**D_2_**) control samples values were set to 100%; in (**B_1_**,**B_2_**) control samples values were set to 1; bars, ± SD (*n* ≥ 2); * *p* < 0.05; ** *p* < 0.01.

Firstly, we examined the effect of SA223-S2BM on the viability of normal skin BJ fibroblasts and human immortalized HaCaT keratinocytes by performing the MTT toxicity assay. For this purpose, cells were incubated for 24 h with increasing concentrations of the extract and, as shown in Figure 2A_1_,A_2_, the extract does not exert a cytotoxic effect on either of the two cell lines examined at concentrations up to 10 μg/mL; at higher concentrations (above 10 μg/mL), the extract reduces cell viability mostly in HaCaT cells.

We then studied the effect of the extract on cellular pathways, which ensure proteome stability of the cell. We observed that short-term (24 h) exposure of BJ fibroblasts to the studied extract significantly induced the expression of the *PSMB7* proteasome gene (at a concentration of 10 μg/mL), while in parallel, it tended to increase the expression of the *PSMB6* gene at the same concentration (Figure 2B_1_). In addition, the extract induced the expression of the 20S proteasome gene, *PSMB6*, and of the 19S proteasome gene, *RPN11* at a concentration of 1 μg/mL in HaCaT cells (Figure 2B_2_). Furthermore, treatment of BJ fibroblasts with the extract for 24 h led to a mild induction of the expression levels of the 20S β5 proteasome subunit at the concentration of 1 μg/mL (Figure 2C). In support of our findings, exposure to the extract significantly increased the chymotrypsin-like proteasome activity both in BJ fibroblasts and HaCaT keratinocytes after 24 h incubation (Figure 2D_1_,D_2_); the different results of the examined concentrations of the extract among BJ and HaCaT cells can be attributed to cell type-specific effects.

**Figure 3:**
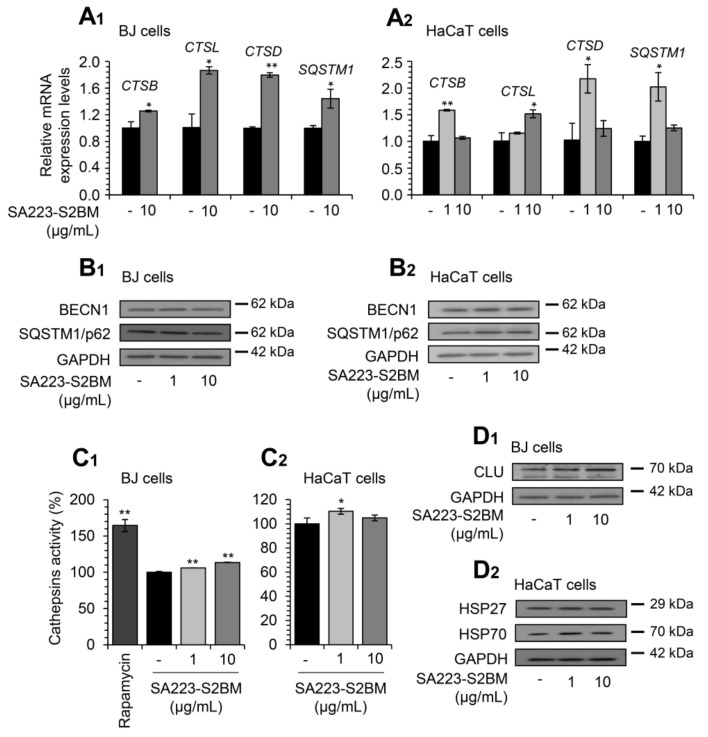
The extract SA223-S2BM activates ALP and induces the expression levels of molecular chaperones. (**A_1_**,**A_2_**) Q-RT-PCR expression analyses of genes involved in ALP functionality (*CTSB*, *CTSL*, *CTSD*, *SQSTM1*) in BJ fibroblasts (**A_1_**) and HaCaT keratinocytes (**A_2_**), after exposure to the indicated concentrations of the SA223-S2BM extract for 24 h. (**B_1_**,**B_2_**) Representative immunoblotting analyses of the autophagy-related proteins BECN1 and SQSTM1/p62 expression levels after treatment of BJ (**B_1_**) and HaCaT (**B_2_**) cells with the shown concentrations of the extract for 24 h. (**C_1_**,**C_2_**) Relative (%) lysosomal-cathepsins activity in BJ (**C_1_**) and HaCaT (**C_2_**) cells, after 24 h treatment with the indicated concentrations of the extract. Rapamycin (100 nM) was used as a positive control. (**D_1_**,**D_2_**) Representative immunoblotting analyses of the molecular chaperones CLU, HSP27, and HSP70 expression levels after treatment of BJ (**D_1_**) and HaCaT (**D_2_**) cells with the shown concentrations of the extract for 24 h. *HMBS* gene expression (**A_1_**,**A_2_**) and GAPDH probing (**B_1_**,**B_2_**,**D_1_**,**D_2_**) were used as references for RNA and protein input, respectively. Quantitation of shown blots is presented in Figures S3 and S4. In (**A_1_**,**A_2_**), control samples values were set to 1; in (**C_1_**_,_**C_2_**), control samples values were set to 100%. Bars, ± SD (*n* ≥ 2); * *p* < 0.05; ** *p* < 0.01.

Additionally, Q-RT-PCR expression analyses revealed that treatment of BJ fibroblasts with the extract for 24 h induced the expression levels of ALP-related genes, named *CTSB*, *CTSL*, *CTSD*, and *SQSTM1*, at a concentration of 10 μg/mL (Figure 3A_1_). Further, we observed that exposure of HaCaT cells to the extract for 24 h led to the induction of the expression levels of genes involved in ALP functionality (*CTSB*, *CTSL*, *CTSD*, and *SQSTM1*) (Figure 3A_2_).

In line with these findings, we noted upregulation of the autophagy-related proteins BECN1 and SQSTM1/p62 after exposure of BJ fibroblasts to the extract at the concentration of 1 μg/mL (Figure 3B_1_); increased expression levels of the proteins BECN1 and SQSTM1/p62 were, also, observed in HaCaT keratinocytes at both studied concentrations (Figure 3B_2_). In accordance, we found that the extract enhanced the lysosomal cathepsins enzymatic activity in both BJ and HaCaT cells (Figure 3C_1_,C_2_); again, the different results of the examined concentrations of the extract among BJ and HaCaT cells can be attributed to cell type-specific effects.

Furthermore, we examined the effect of the extract on key molecular chaperones’ expression levels. Thus, immunoblotting analyses revealed that the extract induced the expression levels of the molecular chaperones CLU (at both studied concentrations) in BJ cells (Figure 3D_1_), as well as of HSP27 (1 μg/mL) and HSP70 (at both studied concentrations) in HaCaT cells (Figure 3D_2_).

### 3.3. Treatment of Flies with the Extract SA223-S2BM Induces Cell Proteostatic Modules Activity

**Figure 4:**
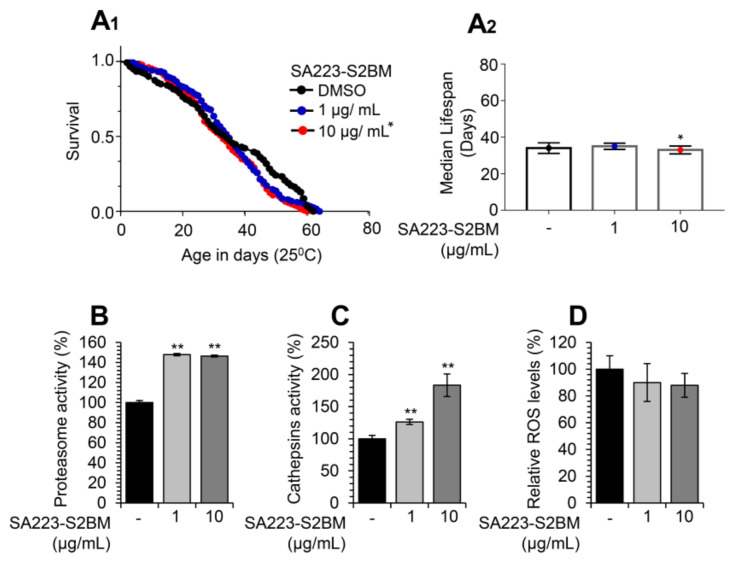
Treatment of *Drosophila* flies with the extract SA223-S2BM activates cell proteostatic modules. (**A_1_**,**A_2_**) Representative longevity curves (**A_1_**) and median lifespan (**A_2_**) of young w^1118^ flies fed with either normal culture medium (DMSO) or with medium supplemented with 1 or 10 μg/mL of the SA223-S2BM extract; comparative statistics are reported in detail in Table S1. (**B**) Relative (%) chymotrypsin-like proteasome peptidase activity in somatic tissues of young (10–13 days of age) w^1118^ flies treated with the indicated concentrations of the SA223-S2BM extract for 10 days. (**C**) Relative (%) cathepsins activity in somatic tissues of young (10–13 days of age) w^1118^ flies treated with the shown concentrations of the extract for 10 days. (**D**) Relative (%) ROS levels in somatic tissues of young (10–13 days of age) w^1118^ flies treated with the shown concentrations of the extract for 10 days. In (**B**–**D**) control samples values were set to 100%; bars, ± SD (*n* ≥ 2); * *p* < 0.05; ** *p* < 0.01.

Considering the observed bioactivity of the SA223-S2BM extract in cell-based assays, we then sought to examine its in vivo effects on the activity of proteostatic modules in the model organism *D. melanogaster* and specifically in w^1118^ *Drosophila* flies. w^1118^ is a transgenic *Drosophila* strain carrying a null mutation in the white gene, an essential gene for the eye pigmentation pathway, and is often used as the appropriate control, because it carries isogenic background for convenient genetic transformations. We found that the extract did not affect flies’ maximum lifespan or healthspan at the concentration of 1 μg/mL, while it marginally decreased flies’ median lifespan at the concentration of 10 μg/mL (Figure 4A_1_,A_2_).

Nevertheless, and in accordance with our findings in human cell lines, administration of the extract in young w^1118^ *Drosophila* flies increased the proteasome chymotrypsin-like (Figure 4B), as well as the lysosomal cathepsins (Figure 4C) enzymatic activities. In addition, as shown in Figure 4D, the extract tended to decrease flies’ tissues ROS levels.

### 3.4. The Extract SA223-S2BM Exhibits Anti-Melanogenic and Anti-Elastase Properties

**Figure 5:**
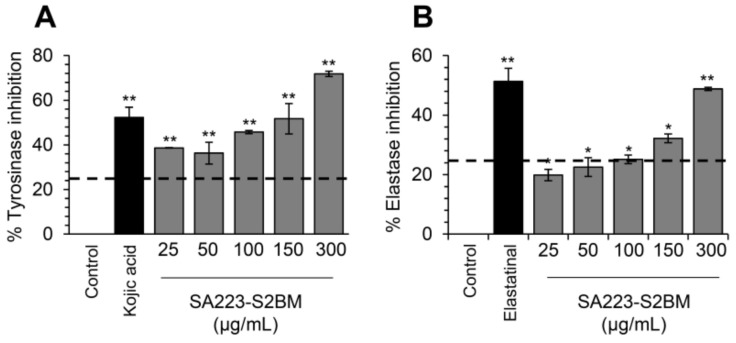
Anti-melanogenic and anti-elastase properties of the SA223-S2BM extract. (**A**) In vitro determination of tyrosinase inhibition activity (%) by the SA223-S2BM extract at the indicated concentrations. (**B**) In vitro evaluation of elastase inhibition activity (%) by the extract at the shown concentrations. In (**A**), Kojic acid (2 μg/mL) was used as a positive control. In (**B**), elastatinal (0.5 μg/mL) was used as a positive control. Bars, ± SD (*n* ≥ 2); * *p* < 0.05; ** *p* < 0.01.

Tyrosinase and elastase are key enzymes implicated in skin aging and their inhibitors are of great importance in the cosmeceutical industry. Thus, we examined whether the extract SA223-S2BM affects the activity of these enzymes by using in vitro spectrophotometric assays. Interestingly, we found that the extract exerted significant anti-melanogenic properties, as it showed a dose-dependently tyrosinase inhibitory capacity, similar to the commercially available tyrosinase inhibitor Kojic acid (Figure 5A). We also observed that the extract presented anti-elastase activity, as compared to the known elastase inhibitor, namely elastatinal (produced by actinomycetes) (Figure 5B).

### 3.5. Incubation of Human Cells with the Extract SA223-S2BM Activates Antioxidant Responses and Protects Cells from Oxidative and Genotoxic Stress

**Figure 6:**
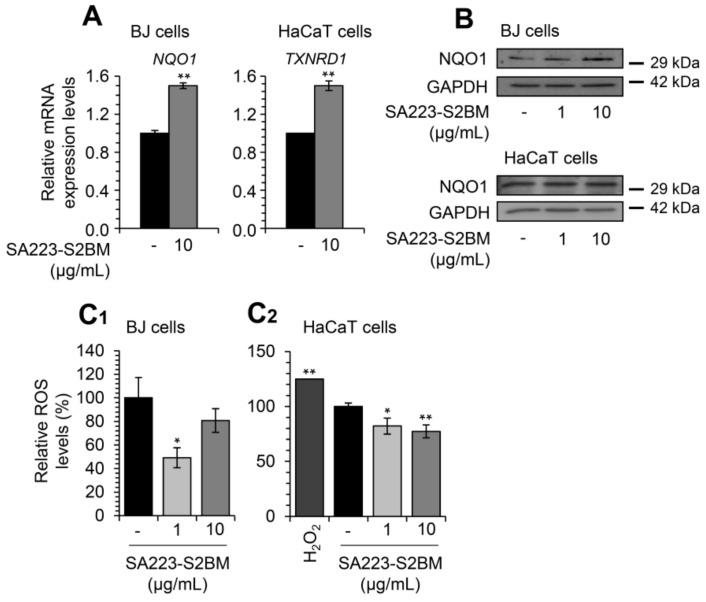
The extract SA223-S2BM activates antioxidant responses-related modules and reduces the intracellular oxidative load. (**A**) Relative expression levels of antioxidant response genes (*NQO1*, *TXNRD1*) in BJ fibroblasts and HaCaT keratinocytes, respectively, treated with the indicated concentration of the extract SA223-S2BM for 24 h. (**B**) Representative immunoblotting analyses of the antioxidant protein NQO1 expression levels after exposure of BJ fibroblast and HaCaT keratinocytes with the shown concentrations of the extract for 24 h. (**C_1_**,**C_2_**) Relative (%) ROS levels after treatment of BJ (**C_1_**) and HaCaT (**C_2_**) cells with 1 and 10 μg/mL of the SA223-S2BM extract for 24 h; shown (%) values represent fluorometry measurement of ROS levels in live cells. Hydrogen peroxide (H_2_O_2_) (200 μM) was used as a positive control. In (**A**), control samples values were set to 1; in (**C_1_**,**C_2_**) control samples values were set to 100%. The *HMBS* gene expression (**A**) and GAPDH probing (**B**) were used as reference for RNA and protein input, respectively. Quantitation of shown blots is presented in Figure S5. Bars, ± SD (*n* ≥ 2); * *p* < 0.05; ** *p* < 0.01.

Then, we investigated the effect of the extract SA223-S2BM on cellular antioxidant modules. We observed that short-term (24 h) incubation of BJ and HaCaT cells with the extract resulted in the upregulation of NRF2 transcriptional targets, named *NQO1* and *TXNRD1* genes, respectively (at the concentration of 10 μg/mL) (Figure 6A). Moreover, we observed by immunoblotting analyses an increase in NQO1 protein expression levels in BJ cells in a dose-dependent manner and in HaCaT cells at the concentration of 10 μg/mL, after exposure to the extract for 24 h (Figure 6B).

Furthermore, we sought to examine whether the extract affects the levels of cellular ROS. Cells were treated with the extract for 24 h and the intracellular ROS levels were evaluated by fluorometry. As shown in Figure 6C_1_,C_2_, the extract significantly decreased ROS levels both in BJ fibroblasts and HaCaT keratinocytes.

**Figure 7:**
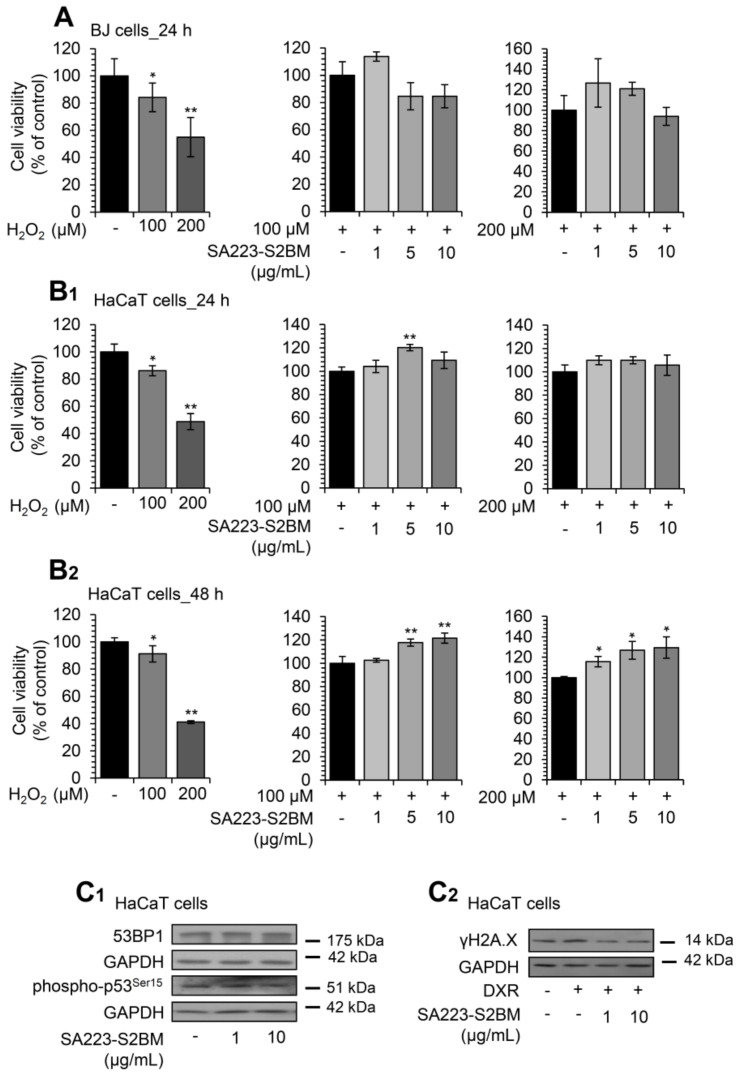
The extract SA223-S2BM confers protection to cells against oxidative and genotoxic stress. (**A**) Relative (%) survival levels (MTT assay) of BJ fibroblasts co-incubated with the oxidative agent H_2_O_2_ (at concentrations of 100 and 200 μM) and the indicated concentrations of the SA223-S2BM extract for 24 h. (**B_1_**,**B_2_**) Relative (%) survival levels (MTT assay) of HaCaT keratinocytes co-incubated with H_2_O_2_ at the concentrations of 100 and 200 μM and the indicated concentrations of the extract for 24 (**B_1_**) and 48 (**B_2_**) h. (**C_1_**) Representative immunoblotting analyses of the expression levels of 53BP1 and the Ser15 phosphorylated form of p53 after exposure of HaCaT cells to the shown concentrations of the extract for 24 h. (**C_2_**) Representative immunoblotting analyses of the Ser139 phosphorylated form of histone H2A.Χ (γH2A.Χ) in HaCaT cells treated for 24 h with 2 μM DXR in the presence or not of the shown concentrations of the extract. In (**A**,**B_1_**,**B_2_**), control samples values were set to 100%. In (**C_1_**,**C_2_**), GAPDH probing was used as reference for protein input. Quantitation of shown blots is presented in Figure S6. Bars, ± SD (*n* ≥ 2); * *p* < 0.05; ** *p* < 0.01.

Subsequently, we investigated whether the extract SA223-S2BM could have a cytoprotective role against oxidative stress-mediated toxicity. Thus, BJ fibroblasts and HaCaT keratinocytes were co-incubated with different concentrations of the extract and the oxidative agent H_2_O_2_, and viability rates were measured after 24 or 48 h of incubation. H_2_O_2_ is well known for its cytotoxic effects [51] and, therefore, was used as a positive control. After a 24 h incubation of BJ cells, we observed that the extract (at the concentration of 1 μg/mL) tended to protect the cells from the toxicity caused by H_2_O_2_ (100 μM); a similar finding was noted after co-treatment of the cells with 200 μM H_2_O_2_ and the extract (1 and 5 μg/mL) (Figure 7A). In support, we found that the extract largely rescued HaCaT cells from the oxidative stress-mediated toxicity, caused by both studied concentrations of H_2_O_2_ (100 and 200 μM), after 24 h (Figure 7B_1_) and 48 h of incubation (Figure 7B_2_).

Additionally, we observed that treatment of HaCaT keratinocytes with the extract for 24 h at a concentration of 10 μg/mL resulted in decreased 53BP1 protein expression levels, as well as of p53 phosphorylation levels at Ser15 in a dose-dependent manner (Figure 7C_1_); both proteins are markers of DNA damage and genotoxic stress [52,53]. The protective effect of the extract against genotoxic stress was also confirmed by immunoblotting analyses after incubating HaCaT cells with the chemotherapeutic drug doxorubicin (DXR). Specifically, we noted that co-incubation of HaCaT cells with the SA223-S2BM extract and 2 μM DXR resulted in the suppression of DXR-induced γH2A.X increased phosphorylation levels in a dose-dependent manner (Figure 7C_2_).

## 4. Discussion

Aging is a process associated with the accumulation of damage and loss of functionality that occur at molecular, cellular, tissue, and organismal level, which relates to increased vulnerability to disease and death [14]. Cellular senescence is a state of permanent and irreversible cell-cycle arrest in response to different damaging stimuli and, among others, is characterized by unique secretory features, macromolecular damage, and altered metabolism [54,55]. Senescent cells accumulate with age, contributing to the development of age-related pathologies [56,57].

It is well established that the activation of PN components could decelerate the rate of aging progression, indicating that these modules may be potential therapeutic targets against aging and age-related pathologies [58,59]. In the current study, we examined at cell-based and in vivo models the effect of the SA223-S2BM extract on proteostatic modules and antioxidant responses (see Graphical Abstract). This extract has been isolated from a soft coral’s symbiotic microorganism namely *S. arenicola*, which is an obligated marine actinomycete belonging to the Micromonosporaceae family [60]. We found that the SA223-S2BM extract activates proteostatic modules both in human cells and *Drosophila* flies.

In addition, the SA223-S2BM extract induced the expression of antioxidant modules, reduced the intracellular oxidative load in skin fibroblasts and immortalized keratinocytes, and conferred protection against oxidative and genotoxic stress. It is evident that cellular senescence and in vivo aging are characterized by increased oxidative stress, increased genomic and proteome instability, as well as by reduced activity of cellular repair mechanisms and proteolytic pathways [14]. Therefore, this extract could be used as a source for the identification of molecules with potential cytoprotective and/or anti-aging properties.

Several studies have shown that both healthspan and lifespan can be improved and extended, respectively, through genetic, nutritional, and/or pharmacological interventions [1]. Indeed, caloric restriction elevates resistance to oxidative stress, reduces macromolecular damage, and increases lifespan in various model organisms [61]. However, as genetic interventions or caloric restriction cannot be applied to humans, many studies have been devoted to the identification of NPs (extracts or isolated compounds) that can potentially increase health- and/or life-span. In support, a variety of NPs, which have been isolated from various sources such as plants, marine organisms, and microorganisms, have been reportedly found to exert anti-aging effects both in vitro and in vivo [1,62].

To date, many marine NPs have been shown to exert a broad range of bioactivities, such as anti-tumor, anti-proliferative, photoprotective, or antibiotic. Therefore, in the past few years, drug discovery from marine NPs has gained ground [63]. Indeed, several marine-derived drugs have already reached the market or are in clinical trials [31]. Very few marine NPs have been studied for their potential to modulate key components of the PN network, and some were found to directly activate UPP and exert anti-aging properties. For instance, Astaxanthin is a xanthophyll carotenoid present in marine organisms, such as macroalgae, microalgae, and crustaceans, and is known for its antioxidant activity both in vitro and in vivo, while it was found to exert anti-aging properties, as it extended lifespan in *C. elegans* and *D. melanogaster* [64].

Tyrosinase and elastase are two key enzymes involved in skin deterioration and therefore, inhibition of their activity is a promising protective method against skin aging [65]. Several NPs derived from plants, e.g., curcumin, have already been studied and found to be effective inhibitors of these enzymes’ activity [66]. Herein, we found that the extract SA223-S2BM inhibited efficiently the activity of tyrosinase and elastase enzymes. Therefore, this extract can be used for the isolation of cosmeceutical products; in support, a great number of marine-derived molecules are already used in the cosmeceuticals industry due to their activity against tyrosinase and elastase [67,68].

As the number of available NPs exerting anti-aging activity is quite low [1], less explored ecosystems, such as the marine ecosystem, need to be further investigated. The marine environment represents a unique resource that encloses a massive biological diversity [30]. Its harsh chemical and physical conditions are important drivers to produce a wide range of bioactive NPs with structurally unique features, which serve as promising templates to inspire drug development against various diseases [31,32]. This is evident in our recent metagenomic analysis, in which the microbial diversity of the marine sponge *Scopalina hapalia* was investigated and the results highlighted the potential of these microorganisms to produce bioactive compounds against age-related molecular targets and, thus, possessing anti-aging properties [36]. However, marine drug discovery must deal with some major challenges, such as structure determination and supply problems [31].

The increased potential of the marine ecosystems to provide bioactive NPs is also confirmed by previous isolations of bioactive products from *S. arenicola*. The most interesting among them is Salinosporamide A (Marizomib), a potent proteasome inhibitor, which entered phase I clinical trials for the treatment of multiple myeloma only three years after its discovery [31,69,70]. Furthermore, the antibiotic Arenimycin, which induces cytotoxicity in human adenocarcinoma cells [71], as well as saliniketal A and saliniketal B, which inhibit ornithine decarboxylase, an anti-cancer target [72], have also been isolated from *S. arenicola*.

## 5. Conclusions

Despite these encouraging findings, the bioactive molecules of the SA223-S2BM extract remain unknown and thus, further investigation is needed. Future studies will reveal the chemical composition of this extract and the bioactive compounds responsible for the extract’s properties, shown in this study. Therefore, we suggest the SA223-S2BM extract, (obtained from an actinomycete of the marine mesophotic coral ecosystem), as a likely effective activator of cytoprotective mechanisms that promote healthy aging.

## Data Availability

Data are available from Corresponding Author upon reasonable request.

## Supplementary Materials

The following supporting information can be downloaded at: https://www.mdpi.com/article/10.3390/cimb44010002/s1, Figure S1: In vitro evaluation of tyrosinase and elastase inhibition activity. (A) (%) Tyrosinase inhibition activity by the SA223-S2BAE extract at the concentration of 150 μg/mL. (B) (%) Elastase inhibition activity by the SA223-S2LAE extract at the concentration of 100 μg/mL. In (A), Kojic acid (2 μg/mL) was used as a positive control. In (B), elastatinal (0.5 μg/mL) was used as a positive control. Bars, ± SD (n≥2); *, *p* < 0.05. Figure S2: Full blots and quantitative analysis of protein expression levels (immunoblots shown in Figure 2). Figure S3: Full blots and quantitative analysis of protein expression levels (immunoblots shown in Figure 3). Figure S4: Full blots and quantitative analysis of protein expression levels (immunoblots shown in Figure 3). Figure S5: Full blots and quantitative analysis of protein expression levels (immunoblots shown in Figure 6). Figure S6: Full blots and quantitative analysis of protein expression levels (immunoblots shown in Figure 7). Table S1: Summary of lifespan experiments. Table S2: Full names of analyzed genes (www.ncbi.nlm.nih.gov/gene).
