# Supplementary material for: Isolation of an Extract from the Soft Coral Symbiotic Microorganism Salinispora arenicola Exerting Cytoprotective and Anti-Aging Effects
Source: Curr Issues Mol Biol. 2021 Dec 22;44(1):14–30. doi: 10.3390/cimb44010002 (PMC8928968; doi:10.3390/cimb44010002)
Supplement: Supplementary file 1 [file cimb-44-00002-s001.zip › Louka_et_al_Table_S1_R1.pdf]

Table S1. Summary of lifespan experiments.

Sample	Genotype	Mean Lifespan (LF) +/- s.e.m. (Days)		Median Lifespan +/- s.e.m. (Days)		% Median LF *vs.* control	*Log Rank P Value*		
							Control	1 µg/mL SA223-S2BM	10 µg/mL SA223-S2BM
Control	w^{1118}	35.111	1.633	34	2.893	100		0.358	0.005
1 µg/mL SA223-S2BM	w^{1118}	37.791	1.290	35	1.683	102.941	0.358		0.236
10 µg/mL SA223-S2BM	w^{1119}	33.133	1.240	33	2.190	97.059	0.005	0.236	

Total Animals Died/Total
117/121
117/120
117/121
