# Supplementary material for: Isolation of an Extract from the Soft Coral Symbiotic Microorganism Salinispora arenicola Exerting Cytoprotective and Anti-Aging Effects
Source: Curr Issues Mol Biol. 2021 Dec 22;44(1):14–30. doi: 10.3390/cimb44010002 (PMC8928968; doi:10.3390/cimb44010002)
Supplement: Supplementary file 1 [file cimb-44-00002-s001.zip › CIMB-1444036-Supplementary.pdf]

current issues in molecular biology

Isolation of an Extract from the Soft Coral Symbiotic Microorganism *Salinispora arenicola* Exerting Cytoprotective and Anti-Aging Effects

Xanthippi P. Louka [1], Aimilia D. Sklirou [1], Géraldine Le Goff [2], Philippe Lopes [2], Eleni-Dimitra Papanagnou [1], Maria S. Manola [1], Yehuda Benayahu [3], Jamal Ouazzani [2] and Ioannis P. Trougakos [1,*]

Supplemental materials

Table S2: Full names of analyzed genes (www.ncbi.nlm.nih.gov/gene).

**Full name**	**Human Gene**
Hydroxymethylbilane synthase	*HMBS*
Proteasome 20S subunit beta 6	*PSMB6*
Proteasome 20S subunit beta 7	*PSMB7*
Proteasome 26S subunit, non-ATPase 14	*PSMD14/ RPN11*
Cathepsin B	*CTSB*
Cathepsin L	*CTSL*
Cathepsin D	*CTSD*
Sequestosome 1	*SQSTM1*
NAD(P)H quinone dehydrogenase 1	*NQO1*
Thioredoxin reductase 1	*TXNRD1*

current issues in molecular biology

MDPI

Supplementary Figures

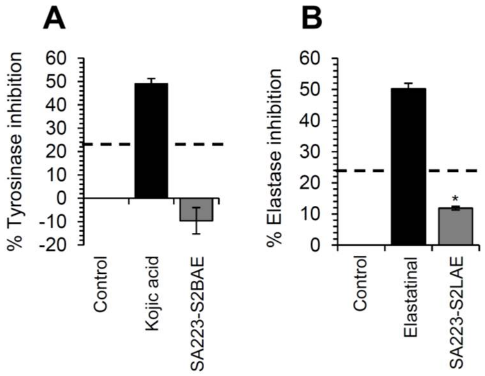

Figure S1. *In vitro* evaluation of tyrosinase and elastase inhibition activity. (A) (%) Tyrosinase inhibition activity by the SA223-S2BAE extract at the concentration of 150 μg/mL. (B) (%) Elastase inhibition activity by the SA223-S2LAE extract at the concentration of 100 μg/mL. In (A), Kojic acid (2 μg/mL) was used as a positive control. In (B), elastatinal (0.5 μg/mL) was used as a positive control. Bars, ± SD (n≥2); *, P < 0.05.

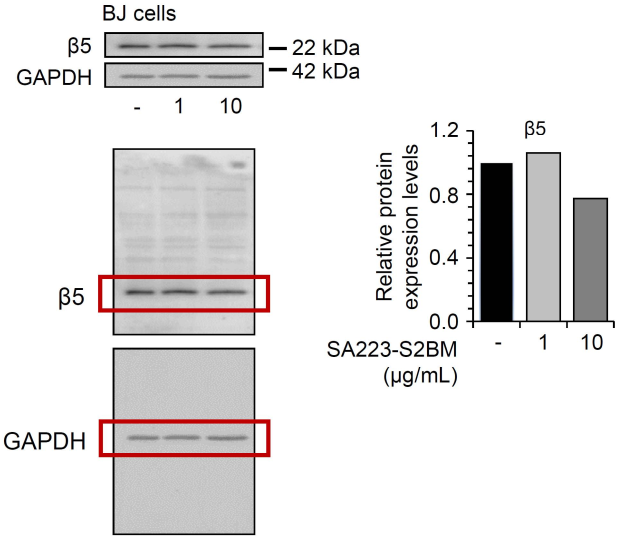

Figure S2. Full blots and quantitative analysis of protein expression levels (immunoblots shown in Figure 2).

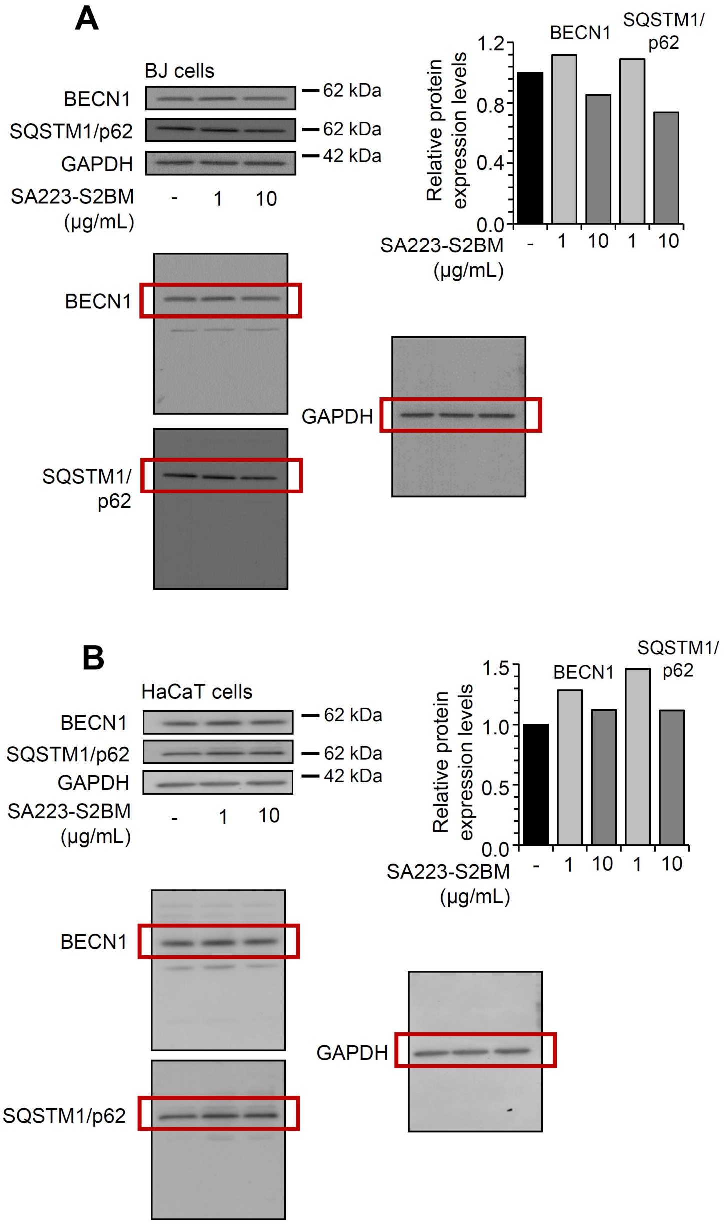

Figure S3. Full blots and quantitative analysis of protein expression levels (immunoblots shown in Figure 3).

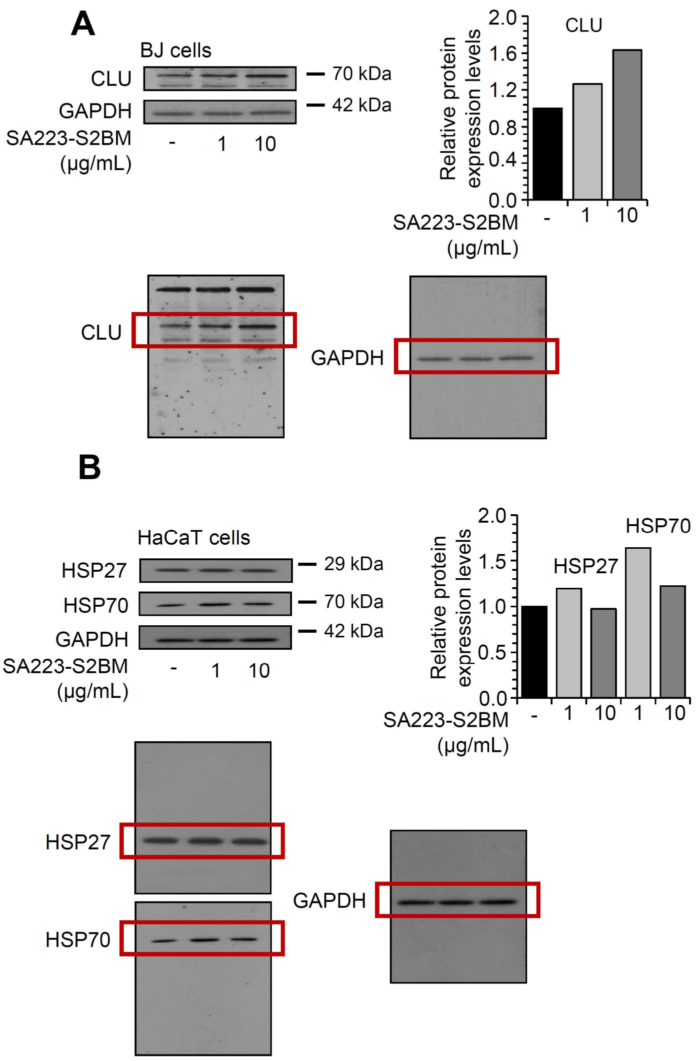

Figure S4. Full blots and quantitative analysis of protein expression levels (immunoblots shown in Figure 3).

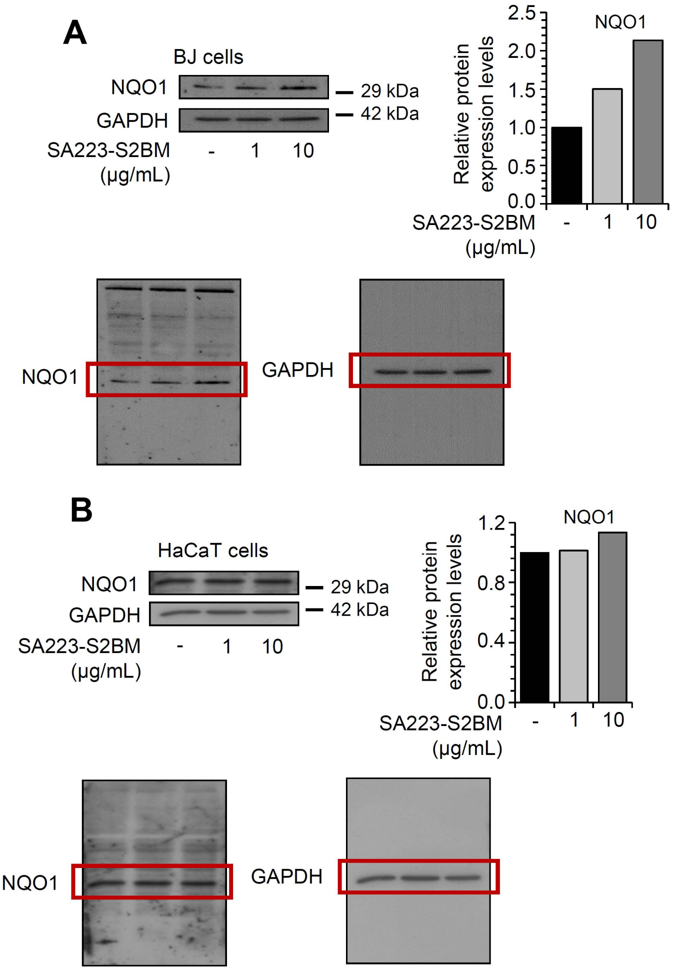

Figure S5. Full blots and quantitative analysis of protein expression levels (immunoblots shown in Figure 6).

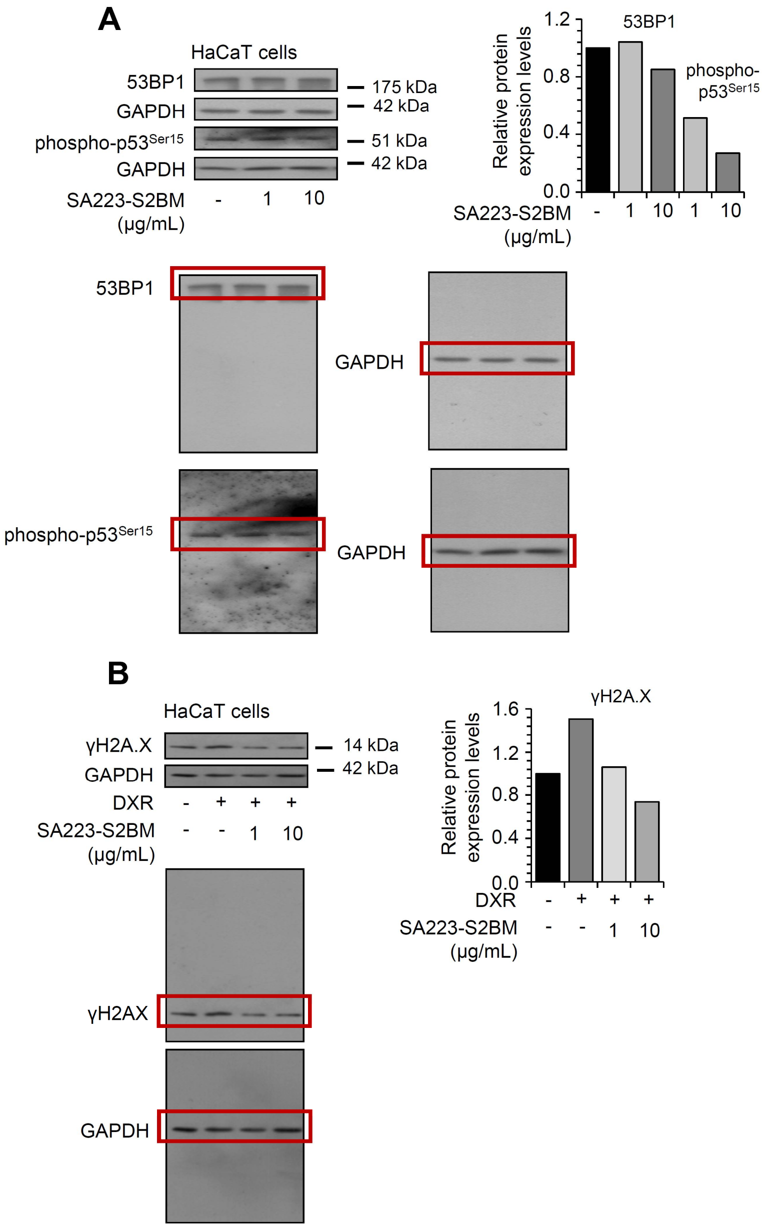

Figure S6. Full blots and quantitative analysis of protein expression levels (immunoblots shown in Figure 7).
